# Karyotype, Sex Determination, and Meiotic Chromosome Behavior in Two Pholcid (Araneomorphae, Pholcidae) Spiders: Implications for Karyotype Evolution

**DOI:** 10.1371/journal.pone.0024748

**Published:** 2011-09-09

**Authors:** Adriana E. Golding, Leocadia V. Paliulis

**Affiliations:** Biology Department, Bucknell University, Lewisburg, Pennsylvania, United States of America; Field Museum of Natural History, United States of America

## Abstract

There are 1,111 species of pholcid spiders, of which less than 2% have published karyotypes. Our aim in this study was to determine the karyotypes and sex determination mechanisms of two species of pholcids: *Physocyclus mexicanus* (Banks, 1898) and *Holocnemus pluchei* (Scopoli, 1763), and to observe sex chromosome behavior during meiosis. We constructed karyotypes for *P. mexicanus* and *H. pluchei* using information from both living and fixed cells. We found that *P. mexicanus* has a chromosome number of 2n = 15 in males and 2n = 16 in females with X0-XX sex determination, like other members of the genus *Physocyclus*. *H. pluchei* has a chromosome number of 2n = 28 in males and 2n = 28 in females with XY-XX sex determination, which is substantially different from its closest relatives. These data contribute to our knowledge of the evolution of this large and geographically ubiquitous family, and are the first evidence of XY-XX sex determination in pholcids.

## Introduction

Spiders display a wide range of chromosome numbers and sex determining systems, and very commonly have multiple X chromosomes. Of the spiders studied, the most common sex determining system is X_1_X_2_0 (male)/X_1_X_1_X_2_X_2_ (female) [1]. Some systems have three or more X chromosomes and/or a Y chromosome (for examples, see [1], [2]).

The spider family Pholcidae currently consists of 84 genera and 1,111 species [3]. Of these species, fewer than 2% have published karyotype data [4]–[16]. The previously studied pholcid species have diploid chromosome numbers between 2n = 15 and 2n = 32, with metacentric or submetacentric chromosomes [4]–[16]. While the majority of spiders have X_1_X_2_0 (male)/X_1_X_1_X_2_X_2_ (female) sex determination, most studied pholcid species have X0 (male)/XX (female) sex determination, though X_1_X_2_ (male)/X_1_X_1_X_2_X_2_ (female) and X_1_X_2_Y (male)/X_1_X_1_X_2_X_2_ (female) sex determination systems have also been observed [4]–[16].

Karyotype data (with information on sex determination) can be helpful in establishing evolutionary relationships between species and for differentiating species that otherwise look similar [2], [17]. In this study we have determined the karyotypes and sex determining systems of two pholcids, *Physocyclus mexicanus* and *Holocnemus pluchei* using observations of living cells and stained fixed cells. We verified our observations of the sex determination mechanism using micromanipulation. We have compared chromosome number and sex determination mechanism with closely related species, and have found that *P. mexicanus* has the same chromosome number and sex determining system as other species of *Physocyclus*, while *H. pluchei* is the first observed example of a pholcid with XY (male)/XX (female) sex determination and has a different karyotype than other closely related species.

## Results

**Figure 1:**
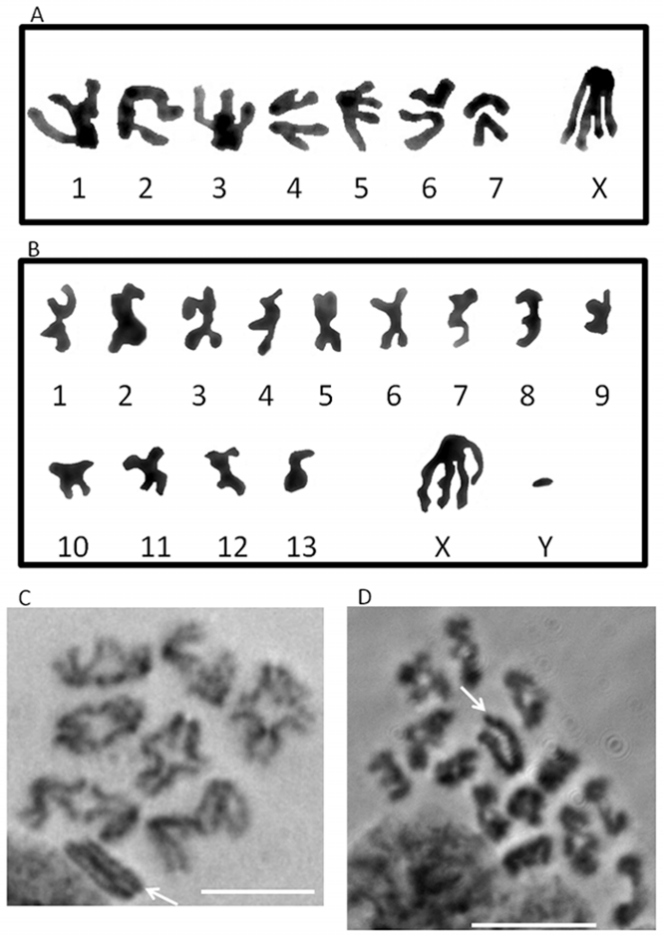
Karyotypes for *Physocyclus mexicanus* and *Holocnemus pluchei* and giemsa-stained meiosis I spermatocytes. A. Haploid karyotype for *Physocyclus mexicanus* taken from fixed, giemsa-stained metaphase II spermatocyte. B. Haploid karyotype for *Holocnemus pluchei* taken from a fixed, giemsa-stained metaphase II spermatocyte (chromosomes 1–13 and Y chromosome) and a fixed, giemsa-stained anaphase I spermatocyte (X chromosome). C. Giemsa-stained metaphase I spermatocyte of *Physocyclus mexicanus*. X chromosome indicated by arrow. Bar = 10 µm. D. Giemsa-stained metaphase I spermatocyte of *Holocnemus pluchei*. X-Y bivalent indicated by arrow. Bar = 10 µm.

*Physocyclus mexicanus* and *Holocnemus pluchei* spermatocytes were observed in metaphase and anaphase of meiosis I and meiosis II to determine chromosome number and sex determination mechanism. Using observations of living and fixed cells, we were able to obtain a karyotype for each species (Figure 1). Karyotypes were constructed using images of Giemsa-stained fixed preparations of cells in anaphase I and metaphase II (chromosomes for karyotypes were obtained from images shown in Figure S1). Preparations from 30 individuals of each species were used to determine karyotypes. In both species, all chromosomes are either metacentric or submetacentric. *P. mexicanus* has a chromosome number of 2n = 15 in males and 2n = 16 in females with X0 (male)/XX (female) sex determination (Figure 1A and 1C). *H. pluchei* has a chromosome number of 2n = 28 in males and 2n = 28 in females with XY (male)/XX (female) sex determination (Figure 1B and 1D).

**Figure 2:**
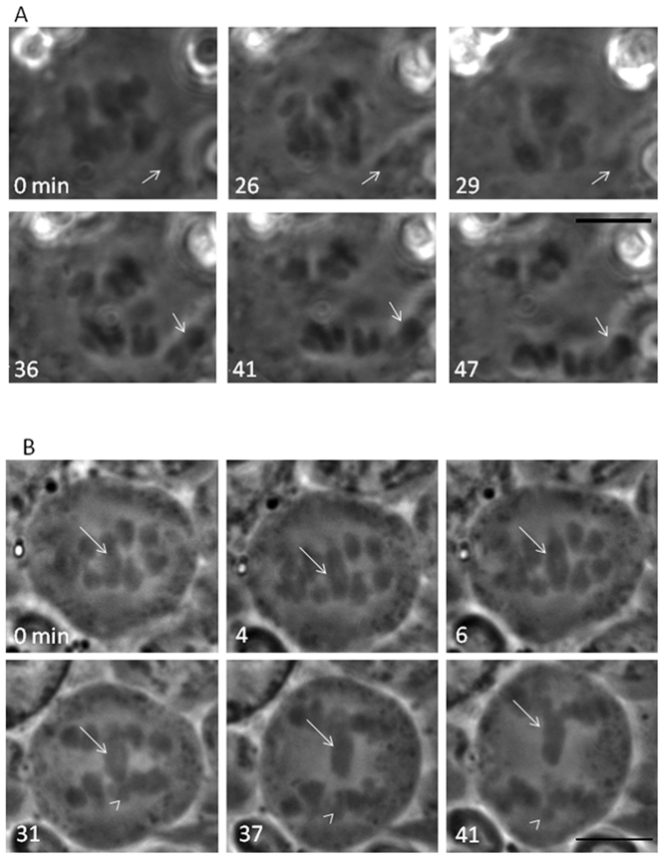
Metaphase I-Anaphase I in living *Physocyclus mexicanus* and *Holocnemus pluchei* spermatocytes. A. Progression from metaphase I (0, 26, 29 min.) through anaphase I (36, 41, 47 min.) in *Physocyclus mexicanus*. A univalent chromosome remains near one spindle pole (arrow) through metaphase I and anaphase I. Bar = 10 µm. B. Progression from metaphase I (0, 4, 6 min.) through anaphase I (31, 37, 41 min.) in *Holocnemus pluchei*. X (arrow) and Y (arrowhead) chromosomes are located in the center of the spindle in metaphase (0, 4, 6 min.) and X and Y chromosomes separate from one another in anaphase (31, 37, 41 min.). Bar = 10 µm.

**Figure 3:**
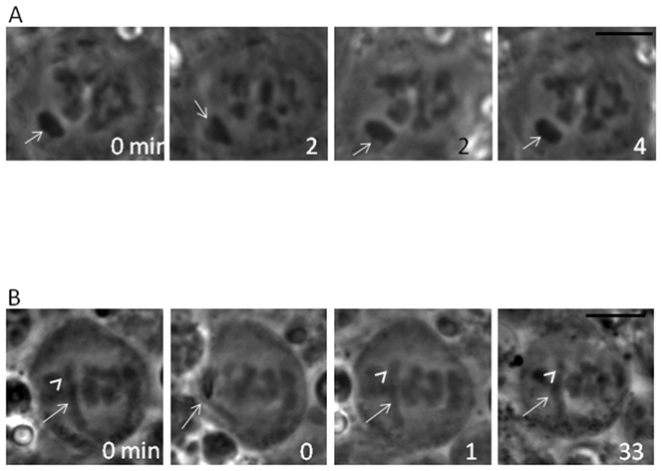
Micromanipulation of primary spermatocytes in *Physocyclus mexicanus* and *Holocnemus pluchei*. A. In a *Physocyclus mexicanus* metaphase I spermatocyte (0 min.), a small micromanipulation needle was used to pull on the univalent chromosome near the spindle pole (2–4 min.), showing a single X chromosome (arrow). Bar = 10 µm. B. In a *Holocnemus pluchei* metaphase I spermatocyte (0 min.), a small micromanipulation needle was used to pull on the sex chromosomes in the middle of the spindle (0 min., arrow). Following the pulling, the chromosomes immediately return to their original position (1, 33 min.), indicating that chromosomes are connected to the spindle at both ends. X chromosome indicated by arrow, Y chromosome by arrowhead. Bar = 10 µm.

Chromosome behavior (including sex chromosomes) was observed in metaphase I and anaphase I spermatocytes of both *P. mexicanus* and *H. pluchei* (Figure 2), in which sex chromosome behavior can be clearly observed. In these studies, we found that *P. mexicanus* males had a univalent X chromosome (Figure 2A, arrows) that remained near one spindle pole from metaphase I through anaphase I. *H. pluchei* males had a large sex chromosome that remained near the center of the spindle (Figure 2B, arrows). Because our previous [18] and current studies (Figure 2A) show that univalent sex chromosomes remain near one spindle pole through metaphase I and anaphase I in spiders, we suspected that the X chromosome was associated with a small Y chromosome, which was apparent in some images (Figure 2b arrowheads). In addition, because we often find that it can be difficult to count the number of X chromosomes present in spermatocytes or to clearly see small Y chromosomes, we also used a small micromanipulation needle to move the sex chromosomes in meiosis I spermatocytes in both species. In organisms with multiple X chromosomes (beyond a single X chromosome, e.g. X_1_X_2_0 (male)/X_1_X_1_X_2_X_2_ (female)—Doan, Andreychik and Paliulis in preparation) it is possible to separate and count the number of sex chromosomes by this technique. Micromanipulation of the sex chromosome in male metaphase I in *P. mexicanus* revealed there was a single X chromosome, showing that males are X0 (Figure 3A, arrows) and confirming X0 (male)/XX (female) sex determination. Micromanipulation experiments were repeated five times, all showing the same result. Micromanipulation of the sex chromosomes in *H. pluchei* showed that the sex chromosome is not a univalent, but a bivalent, as applying tension on the center of the chromosome (Figure 3B, arrows) showed that there are two spindle-attachment points on the chromosome. Further, we can visualize the very small Y chromosome in some images (Figure 2B and 3B, arrowheads). This micromanipulation experiment was performed five times, always showing that there are two spindle attachment sites on the sex chromosomes, and confirming XY (male)/XX (female) sex determination. Our data show that *P. mexicanus* has chromosome number of 2n = 15 in males and 2n = 16 in females, and that *H. pluchei* has a chromosome number of 2n = 28 in males and 2n = 28 in females (Figure 3).

## Discussion

Our data show that *Physocyclus mexicanus* has a karyotype that is very similar to that of other species of *Physocyclus*. Previously published studies reveal that *Physocyclus globosus* [5], *Physocyclus californicus*, and *Physocyclus enaulus* [9] have 2n = 15 = 14+X in males and 2n = 16 = 14+XX in females, like we saw with *P. mexicanus*. All *P. mexicanus*, chromosomes appear to be metacentric or submetacentric, as observed in *P. globosus* [5], *P. californicus*, and *P. enaulus* [9].

Our study of *Holocnemus pluchei* reveals a chromosome number of 2n = 26+XY in males and 2n = 26+XX in females. Král et al. [6] showed that another species of *Holocnemus*, *H. caudatus* has a chromosome number of 2n = 22+X in males and 2n = 22+XX in females. *H. caudatus* and *H. pluchei* have different numbers of autosomes but they do have chromosomes that appear to have similar morphology, as all chromosomes in both species appear to be either metacentric or submetacentric (this study, [6]). Sex determination is different between species, with *H. caudatus* having XO-XX sex determination, and *H. pluchei* having XY-XX. Both *H. caudatus* and *H. pluchei* have very long metacentric X chromosomes (X is the longest chromosome in the karyotypes of both species) that appear very similar in morphology (this study, [6]). Another very closely related species (based on molecular phylogeny using multiple gene regions—[19]), *Crossopriza lyoni* appears to have different karyotypes in different populations around the world. The population of *C. lyoni* studied by Oliveira et al. [5] appears to have a very similar karyotype, with similar autosome morphology and sex determination mechanism to *H. caudatus*. It also has similar autosome and X-chromosome morphology to *H. pluchei*, but, like *H. caudatus*, *C. lyoni* also lacks several autosomes and the Y chromosome we observe in *H. pluchei*. Other populations of *C. lyoni* have different numbers of autosomes and sex determining mechanisms [10]–[13], but none have the same number of autosomes or the same sex determining mechanism as *H. pluchei*.

As stated previously, this is the first published demonstration of an XY-XX sex determination system in pholcids, though X_1_X_2_Y (male)/X_1_X_1_X_2_X_2_ (female) systems have been observed several times [6]. Král et al. [6] proposed a mechanism for the evolution of sex determining systems from an X_1_X_2_Y (male)/X_1_X_1_X_2_X_2_ (female) system to an X0 (male)/XX (female) system in primitive araneomorph spiders (e.g. pholcids), in which one of the intermediates is an XY (male)/XX (female). In this proposed mechanism, the ancestral form is an X_1_X_2_Y (male)/X_1_X_1_X_2_X_2_ (female) system, which is observed in the pholcid *Spermophora senoculata*. According to the phylogeny of pholcids constructed by Bruvo-Mađarić et al. [19], *Spermophora senoculata* is basal to *Holocnemus pluchei*. In *S. senoculata*, both X_1_ and X_2_ are metacentric and there is a very small metacentric Y chromosome [6]. Král *et al.* proposed that both X_1_ and X_2_ are converted from metacentric chromosomes to acrocentric chromosomes by pericentric inversions [6]. Then, a Robertsonian translocation between X_1_ and X_2_ forms a single metacentric X chromosome [6]. Král mentions an XY (male)/XX (female) sex determining system in the pholcid *Smeringopus pallidus* as unpublished data [6]. *Smeringopus pallidus*, is basal to *Holocnemus pluchei* on the phylogenetic tree constructed by Bruvo-Mađarić et al. [19], potentially explaining the presence of an XY (male)/XX (female) sex determining system in *H. pluchei*. Král et al. proposed that the small Y chromosome is lost in some lineages, leading to an X0 (male)/XX (female) sex determining system in Holocnemus caudatus.

Based on morphological characters, the genera *Holocnemus* and *Physocyclus* were placed in the subfamily Holocneminae [19], [20]. However, recent molecular phylogenetic data show that they are far more distantly related than initially thought [19], [20], which is supported by the significant differences in the karyotypes of *Holocnemus* and *Physocyclus* (i.e. large differences in chromosome number and morphology). Our current results in comparison with the previously obtained results of closely related species show that closely related species have similar chromosome number and structure (e.g., the different species of *Physocyclus*), but that key changes can happen concomitant with or following speciation, as we have deduced by comparing chromosome number and sex chromosome behavior in *Holocnemus pluchei* with the previously obtained karyotypes of *Holocnemus caudatus* and *Crossopriza lyoni* [5], [6]. In addition, in *H. pluchei* we have found the first evidence of an XY-XX sex determination system in pholcids. Further analysis will be required to determine whether the hypothesis of Král *et al*. explains the evolution of the sex determining system of *Holocnemus pluchei*. In addition, further study will be necessary to explain why *H. pluchei* has more autosomes than *H. caudatus* and *C. lyoni*, its two closest relatives.

These results add to the known karyotype information for the family Pholcidae, allowing further understanding of karyotype evolution in this family. When chromosome data for other pholcids are obtained, these results have the potential to elucidate the phylogeny for this family.

## Materials and Methods

Living *Physocyclus mexicanus* and *Holocnemus pluchei* males and females were obtained from Spider Pharm Inc. (Yarnell, AZ). Spiders were collected in Yarnell, AZ, USA and identified by C. Kristensen. The authors verified the identification. Specimens are deposited in the National Museum of Natural History, Smithsonian Institution.

### Giemsa staining of chromosomes

Adult *Physocyclus mexicanus* and *Holocnemus pluchei* testes were fixed in 6:3:1 ethanol:chloroform:acetic acid for 10 minutes, testes were macerated in 45% acetic acid and pipetted using a pasteur pipet to produce a cell suspension. The cell suspension was spread on a microscope slide and placed at 60°C until the cell suspension had dried. Chromosomes were stained with 5% giemsa for 5 minutes, mounted, and observed using a Zeiss inverted microscope.

### Living cell preparations

Living cell preparations of adult male testes were prepared at room temperature according to the method of Doan and Paliulis [18]. Primary and secondary spermatocytes undergoing meiosis were filmed across multiple focal planes. To verify sex determination method, micromanipulation was used to position sex chromosomes in meiosis I spermatocytes so the number of pairs of sister chromatids could be determined. Tension was applied to determine whether meiosis I sex chromosomes were univalent or bivalent [18].

## Supporting Information

Figure S1 **Chromosome spreads used to derive karyotypes in** **Figure 1** **.** A. Giemsa-stained spread of *Physocyclus mexicanus* metaphase II spermatocyte used to derive karyotype in Figure 1A, with eight chromosomes. Arrow points to X chromosome. Bar = 10 µm. B. Giemsa-stained spread of *Holocnemus pluchei* metaphase II spermatocyte used to derive all chromosomes but X chromosome in karyotype in Figure 1B, with 14 chromosomes. Arrowhead points to Y chromosome. Bar = 10 µm. C. Giemsa-stained spread of *Holocnemus pluchei* anaphase I spermatocyte used to derive X chromosome in karyotype in Figure 1B, with 28 chromosomes. Arrow points to X chromosome. Arrowhead points to Y chromosome. Bar = 10 µm.
(TIF)
