# Supplementary figures and images for: Karyotype, Sex Determination, and Meiotic Chromosome Behavior in Two Pholcid (Araneomorphae, Pholcidae) Spiders: Implications for Karyotype Evolution
Source: PLoS One. 2011 Sep 9;6(9):e24748. doi: 10.1371/journal.pone.0024748 (PMC3170389; doi:10.1371/journal.pone.0024748)

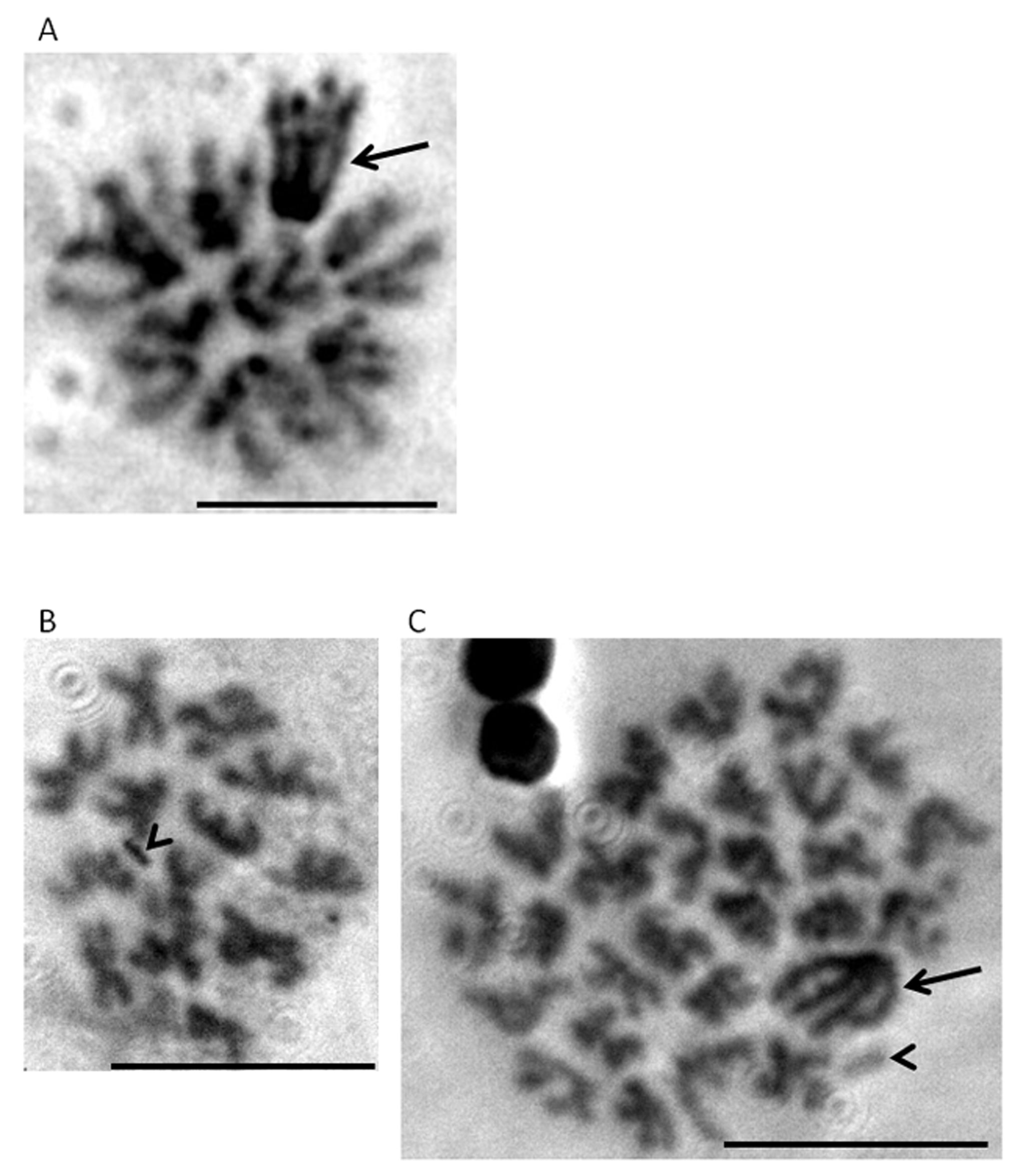

Supplement: Figure S1 — Chromosome spreads used to derive karyotypes in Figure 1 . A. Giemsa-stained spread of Physocyclus mexicanus metaphase II spermatocyte used to derive karyotype in Figure 1A, with eight chromosomes. Arrow points to X chromosome. Bar = 10 µm. B. Giemsa-stained spread of Holocnemus pluchei metaphase II spermatocyte used to derive all chromosomes but X chromosome in karyotype in Figure 1B, with 14 chromosomes. Arrowhead points to Y chromosome. Bar = 10 µm. C. Giemsa-stained spread of Holocnemus pluchei anaphase I spermatocyte used to derive X chromosome in karyotype in Figure 1B, with 28 chromosomes. Arrow points to X chromosome. Arrowhead points to Y chromosome. Bar = 10 µm. (TIF) [file pone.0024748.s001.tif]
